# Oral Anticoagulation in Patients with Chronic Liver Disease

**DOI:** 10.3390/medicina59020346

**Published:** 2023-02-12

**Authors:** Raluca S. Costache, Andreea S. Dragomirică, Bogdan E. Gheorghe, Vasile D. Balaban, Silviu M. Stanciu, Mariana Jinga, Daniel O. Costache

**Affiliations:** 1 Internal Medicine and Gastroenterology Discipline, Carol Davila University of Medicine and Pharmacy, 7000 Bucharest, Romania; 2 Gastroenterology Department, Carol Davila University Central Emergency Military Hospital, 7000 Bucharest, Romania; 3 Dermatology II Discipline, Carol Davila University of Medicine and Pharmacy, 7000 Bucharest, Romania

**Keywords:** liver disease, VKAs, DOACs, atrial fibrillation, venous thrombosis

## Abstract

The administration of an anticoagulant in patients with liver disease (nonalcoholic steatohepatitis—NASH, nonalcoholic fatty liver disease—NAFLD, chronic hepatitis, or cirrhosis) who have an indication (atrial fibrillation, venous thrombosis, or pulmonary embolism) is challenging because there is an imbalance between thrombosis and bleeding. There is a need to focus our attention on preventing risk factors because diabetes, obesity, dyslipidemia, smoking, and sedentary behavior are risk factors for both NASH/NAFLD and AF, and these patients require anticoagulant treatment. Patients with advanced liver disease (Child–Pugh C) were excluded from studies, so vitamin K antagonists (VKAs) are still recommended. Currently, VKAs are recommended for other conditions (antiphospholipid syndrome, mitral valve stenosis, and mechanical valve prosthesis). Amongst the patients under chronic anticoagulant treatment, especially for the elderly, bleeding as a result of the improper use of warfarin is one of the important causes of emergency admissions due to adverse reactions. DOACs are considered to be efficient and safe, with apixaban offering superior protection against stroke and a good safety profile as far as major bleeding is concerned compared to warfarin. DOACs are safe in the Child–Pugh A and B classes (except rivaroxaban), and in the Child–Pugh C class are contraindicated. Given that there are certain and reliable data for chronic kidney disease regarding the recommendations, in liver function impairment more randomized studies must be carried out, as the current data are still uncertain. In particular, DOACs have a simple administration, minimal medication interactions, a high safety and effectiveness profile, and now a reversal agent is available (for dabigatran and idarucizumab). Patients are also statistically more compliant and do not require INR monitoring.

## 1. Introduction

In the USA, thrombosis is a conductive factor in increased morbidity and mortality and it is involved in the etiology of important disorders, especially in ischemic stroke, venous and/or arterial thromboembolism (VTE), and cardiac arrest. For many years, warfarin has been used for the management and prophylaxis of venous thrombosis and ischemic stroke in patients with atrial fibrillation (AF), which is the most frequent cardiac rhythm disorder [1]. After 50 years, warfarin continues to be one of the most common oral anticoagulants (OAC) for VTE, stroke prevention in patients with AF, and for patients with mechanical heart valves [2]. However, an observational study of more than three million patients with non-valvular atrial fibrillation concluded that VKA use had declined from 2010 to 2017, while DOAC use had significantly increased in those patients [3]. One of the major causes of liver function impairment is nonalcoholic fatty liver disease (NAFLD), often leading to cirrhosis and hepatocarcinoma and is an important risk factor for cardiovascular disease because it fits well among the cardiometabolic risk factors, such as smoking, obesity, dyslipidemia, and diabetes mellitus. The association between AF and NAFLD (because they have the same risk factors) is common, but using OAC in liver pathology is complicated because of the lack of evidence; most of them have been disqualified from clinical trials. The number of patients with NAFLD or nonalcoholic steatohepatitis (NASH) who require anticoagulation is steadily increasing, making them perfect subjects for additional investigation examining the effects of long-term anticoagulation, particularly direct oral anticoagulants (DOACs), on fibrogenesis and portal hypertension.

However, NAFLD is strongly associated with metabolic syndrome and it is a problem distinguishing which of both entities independently contribute to a procoagulant state. It seems that NASH, the necro-inflammatory form of NAFLD, contributes significantly and independently to procoagulant and prothrombotic states. Therefore, this evidence shows the benefits of the assessment of the prothrombotic and procoagulant risk factors in chronic liver diseases, particularly in NAFLD [4].

Some studies have demonstrated the association between NAFLD and the risk of stroke. It seems that the risk of stroke gradually increases with an increase in the fatty liver index, which is why patients with NAFLD should be counseled and carefully monitored for the risk of stroke [5]. Additionally, other studies have shown that NAFLD was independently associated with a higher risk of incident AF, which is accentuated in individuals with low and normal weight [6].

Papatheodoridis et al. demonstrated in their study that thrombotic risk factors are frequently detected in patients with chronic viral hepatitis and more extensive fibrosis and advanced stages are associated with at least one of the significant factors [7].

Regarding patients with cirrhosis, DOACs can be used safely except for in Child–Pugh C cirrhosis. Portal vein thrombosis (PVT), one of several indicators of progressive liver disease, is a common complication in cirrhosis that needs anticoagulation. Another indication for anticoagulation therapy in these patients is the prevention or treatment of liver fibrosis [8,9].

This review presents the safety and potency of DOACs in mild–moderate hepatic impairment liver function. Even though the actual guidelines recommend DOACs in chronic liver disease, there is not sufficient data, which is why more perspective and solid reports are needed [10].

## 2. Indications for Oral Anticoagulation

The general evidence for prescribing OAC are the following: valvular and non-valvular atrial fibrillation for the prevention of ischemic stroke and mechanical valve (indication for using warfarin with an international normalized ratio (INR) target between 2–3, increased in mechanical valves). For nonvalvular atrial fibrillation, deep vein thrombosis, pulmonary embolism, and VTE prophylaxis in orthopedic surgery DOACs or warfarin are used [11].

Management of anticoagulated patients with liver disorders is difficult because of an increased risk of bleeding (correlated with a reduction in synthetic liver functions in cases of progressive liver disease, varicose lesions, and thrombocytopenia) and also a higher ischemic risk. In recent research, patients with AF with liver cirrhosis had no improvement in hemorrhagic events on using DOACs in contrast with VKAs. DOACs are not recommended for patients with Child–Pugh cirrhosis, and rivaroxaban is contraindicated for Child–Pugh B or C patients [12]. Apixaban and rivaroxaban have shown pharmacokinetic characteristics similar to non-hepatically unhealthy patients for individuals with Child–Pugh Class A and B. Each DOAC has a different hepatic excretion rate (20 percent for dabigatran, 65 percent for rivaroxaban, 50 percent for edoxaban, and 75 percent for apixaban), but warfarin has a 100 percent hepatic excretion rate, implying more predictable pharmacokinetics for DOACs in liver cirrhosis [10,13]. In addition, INR is a less accurate monitoring indicator in hepatic dysfunction due to metabolic alterations in coagulation factors synthesis. Despite being hypercoagulable, cirrhotic individuals have elevated serum INRs, which could lead to a misleading identification of therapeutic warfarin anticoagulation despite sub-therapeutic dosages. Even though these obstacles appear to favor DOACs, anticoagulation with warfarin is still preferable in hepatic dysfunction due to its ability to be managed and adjusted appropriately, in contrast to DOACs, which lack a defined and proven to-measure criterion [10].

## 3. Warfarin in Liver Disease

Participants with hepatic disease had higher risks of ischemic stroke and deep venous thrombosis than patients without liver disease, according to some observational studies throughout the general population [14]. The presence of liver cirrhosis was independently related to a greater risk of ischemic stroke in a retrospective study of 289,559 individuals with AF from Taiwan’s National Health Insurance Research Database, compared to patients without liver cirrhosis [15].

Furthermore, a meta-analysis of nine observational studies indicated that individuals with liver disease have a 2.5-fold increased risk of ischemic stroke than those without liver disease [16]. Ischemic stroke in individuals with liver function impairment is a predictor of poor prognosis and is linked to a greater risk of mortality in the hospital.

Patients with chronic liver disease have an increased risk of portal vein thrombosis (PVT) due to poor flow in the splanchnic veins, intra-abdominal infections, malignancy, inflammation, and compression from secondary splenomegaly and ascites, concerning the risk of deep vein thrombosis and pulmonary embolism. PVT is the most common thrombotic presentation in individuals with liver damage, according to research, with a frequency of 8% to 18% in patients with liver cirrhosis, and it is a sign of poor outcomes. PVT developed in 11% of subjects after 5 years and was linked to the existence of advanced liver disease symptoms such as esophageal varices and hepatic coagulopathy [17].

Even with its limited therapeutic range, warfarin is difficult to employ in regular medical care, especially in patients with chronic liver disease. However, due to its metabolism via the cytochrome P450 enzymes, warfarin is susceptible to severe drug–drug interactions, resulting in supra-therapeutic or sub-therapeutic international normalized ratio (INR) levels. This trait can be harmful in patients with hepatic dysfunction, who are at a higher risk of bleeding and thrombosis.

When compared with individuals without liver diseases, patients with liver disease had a shorter mean duration in the therapeutic range, which has been linked to a 2-fold increase in the risk of bleeding. In individuals with liver illness, additional parameters such as albumin and glomerular filtration rate may have a major impact on this incidence [18].

Warfarin was linked to a 24 percent decreased incidence of ischemic stroke when compared to no antithrombotic medication in a statistical model study of 10,336 individuals with liver fibrosis and a CHA2DS2-VASc score of 2 in Taiwan [15].

Early administration of warfarin was substantially linked to recanalization of the portal vein in a case study of 55 individuals with cirrhosis and secondary PVT. On the other hand, anticoagulation with warfarin was linked to gastrointestinal hemorrhage in five individuals (9%) due to the larger varices [19].

Even though warfarin has been shown in randomized trials of patients with AF and VTE to reduce the risk of thromboembolism in patients with impaired liver function, it has several limitations in medical practice, including the need for frequent INR measurement, interactions with food and prescription drugs, phenotypic variation in reaction, and increased rates of intracerebral hemorrhage and death, especially in contrast to DOACs [10].

Even apart from patients with liver diseases, bleeding as a result of warfarin’s improper use is one of the important causes of emergency admissions due to adverse reactions among patients under chronic anticoagulant treatment, especially in the elderly [20,21].

## 4. DOACs in Liver Disease

Patients with hepatic function impairment may not be the optimal candidates for these medicines since there is no good monitoring measure to check indicators for their safety. The Child–Pugh grading system and selection criteria used in pivotal studies are used to limit the use of DOACs in individuals with hepatic impairment. In patients with severe hepatic disease, all DOACs are contraindicated, and warfarin is the only anticoagulant approved for these patients [22]. In patients with modest hepatic impairment, dabigatran, apixaban, and edoxaban are acceptable alternatives that do not require dosage changes. Due to a lack of data, the best anticoagulation approach for these patients is unknown, hence blood tests to assess liver function and coagulation parameters should be obtained before starting and frequently following DOAC medication [23].

### Recommendations for the DOACs Due to the Severity of Liver Disease

For Child–Pugh A, there is no dosage reduction for dabigatran, rivaroxaban, apixaban, edoxaban, or betrixaban. Dabigatran, apixaban, edoxaban, and rivaroxaban should be used with attention to usage for Child–Pugh B (betrixaban—not applicable (NA)), and contraindication for all of these drugs is indicated in Child–Pugh C (score more than 9) (rivaroxaban is contraindicated in Child–Pugh B) [23], according to the 2021 European Heart Rhythm Association Practical Guide for DOACs (Table 1).

Most DOACs are exposed to some extent of liver metabolism, particularly cytochrome p450 enzymes in the case of some DOACs. As a result, compromised liver functions are thought to increase drug levels and hemorrhage risks.

Patients with current or chronic liver illness were often excluded from large-scale randomized controlled trials (RCTs). In landmark RCTs, anemia and thrombocytopenia, both of which are likely to be present in CLD, were also ruled out.

In the ROCKET AF trial, for rivaroxaban, the exclusion criteria (indicators) were acute or chronic hepatitis, liver cirrhosis, an ALT > 3x upper limit of normal (ULN), or a level of hemoglobin < 10 g/dL [24].

In the ARISTOTLE trial, for apixaban, the exclusion criteria conditions were AST or ALT 2x ULN or total bilirubin > 1.5x ULN, hemoglobin < 9 g/dL, or thrombocytopenia < 100.000/mm^3^ [25].

In the RE-LY trial, dabigatran was ruled out with persistently high levels of ALT or AST, the presence of hepatitis A, B, or C, anemia (hemoglobin < 10 g/dL), or thrombocytopenia < 100.000/mm^3^ [26].

In the ENGAGE AF-TIMI 48 trial, when ALT or AST > 2x UL or total bilirubin > 1.5 UL, anemia (hemoglobin under 10 g/dL), or thrombocytopenia (100.000/m^3^), precaution is recommended for edoxaban [27].

## 5. OACs Medication in Liver Disease

Before starting OACs, all patients with or at risk of liver disease might have their liver function tests, platelet levels, serum creatinine, and coagulation profile evaluated, and the results should be monitored during therapy. In the context of significant thrombocytopenia (platelet count levels between 50,000 and 70,000/mm^3^), anticoagulation medication should be postponed, based on the patient’s thrombotic risk [28].

Before administering OACs, all at-risk patients must be examined for varices and high-risk abnormalities. Before starting OAC, all individuals with liver disease should be evaluated for alcohol abuse and, if necessary, they should be provided with cessation therapy [29].

Patients with a significant recent bleeding event, persistent coagulopathy, or clinically significant hemorrhagic risk (including high-risk esophageal varices) should be given personalized anticoagulant therapy. Even though warfarin has generally been the therapeutic option for most patients with hepatic impairment who need OACs, DOACs (without dosage adjustment) might be a safe option in certain individuals with minimal hepatic impairment (Child–Pugh A).

In patients with serious hepatic impairment, warfarin seems to be the only OAC that is advised (Child–Pugh C). When warfarin is not an option, apixaban, dabigatran, or edoxaban may be cautiously administered in individuals with mild hepatic impairment (Child–Pugh B). Early collaboration between cardiologists and gastroenterologists should assist in the optimal use of OACs in patients with chronic liver disease who are already at high risk of bleeding and thrombosis at the same time [10].

Cirrhotic patients, irrespective of etiology balance between a hypercoagulable and a hypercoagulable state, are often associated with conditions that require antithrombotic treatment for prophylactic or therapeutic purposes. Therefore, the clinician often faces serious challenges in establishing if anticoagulant therapy would bring the patient benefits that outweigh the bleeding risks. Practice guidelines developed by relevant scientific organizations try to facilitate the process. For example, the AGA suggests standard anticoagulation prophylaxis in patients with cirrhosis and who otherwise meet standard guidelines for the use of VTE prophylaxis over no anticoagulation [30].

Special concerns regarding anticoagulation have been introduced involving patients with portal vein thrombosis (PVT) and concomitant liver disease [31]. The American Gastroenterological Association Institute (AGA) suggests against PVT routine screening in cirrhotic patients, except for candidates for liver transplantation [30].

Historically, research on this topic has been focused on unfractionated heparin, low molecular weight heparin, and AVKs, but recently there has been increasing interest in evaluating DOACs as well, thus generating greater complexity of choice between the available pharmacological agents.

The findings are that the anticoagulated patients with cirrhosis have similar non-variceal bleeding complication risk to the general population and their bleeding risk related to portal-hypertensive complications remains unchanged [31]. However, decisions about initiating anticoagulant therapy should be made in an individualized fashion. Screening esophagogastroduodenoscopy (EGD) for the diagnosis of esophageal and gastric varices is recommended when the diagnosis of cirrhosis is made (Class IIa, Level C), so that assessment of high bleeding risk lesions related to portal hypertension is inherently made [29].

However, although recanalization of the portal axis has been observed to occur spontaneously in some patients with cirrhosis who develop PVT, there are studies that show anticoagulation improves the recanalization rate (42% of patients with anticoagulation therapy alone and 13% of patients who did not receive anticoagulation or vascular intervention) [31]. Furthermore, the AGA suggests using anticoagulation over no anticoagu-lation for the treatment of PVT, citing a lack of data supporting one anticoagulant over another [30].

A beneficial approach seems to be waiting a period of time to identify patients with progressive or persistent PVT before starting anticoagulants. The optimal time for initiation of therapy is described as being less than 6 months, but individuals may benefit from earlier initiation as well. In patients with cirrhosis who have recent thrombosis of small intrahepatic sub-branches of the PV or minimally occlusive (<50% obstruction of the lumen) thrombosis of the main PV, observation with serial imaging every 3 months without therapy is reasonable, while recent occlusion or partial occlusion (>50% of the lumen occluded) requires treatment in order to avoid the progression of portal hypertension and complicate future liver transplantation. Cavernous transformation of the portal vein with the development of collaterals does not require anticoagulant. The duration of therapy with traditional anticoagulation is unclear, and dosing is not standardized.

Whenever candidates for liver transplantation are involved, anticoagulant therapy may be initiated with the goal of recanalization as a patent main portal vein is associated with an increased posttransplant survival rate [31,32].

## 6. Pharmacology of OACs in Liver Disease

In the general subset of patients with AF and VTE, DOACs have been demonstrated to decrease stroke and thrombosis safely. Patients with liver impairment, on the other hand, have been mostly eliminated from randomized clinical studies of OACs for stroke and venous thromboembolism (VTE) prevention. Additionally, because all presently authorized DOACs require extensive hepatic metabolism, liver dysfunction may result in higher drug levels, lower coagulation factor levels, and higher bleeding risks. Furthermore, the metabolism of several DOACs is dependent on cytochrome P450 enzymes [33], and the activity of these enzymes may be altered or changed in liver disease. As a result, the best anticoagulation approach for individuals with AF and VTE who also have liver function impairment is complicated and poorly characterized [10].

**Figure 1:**
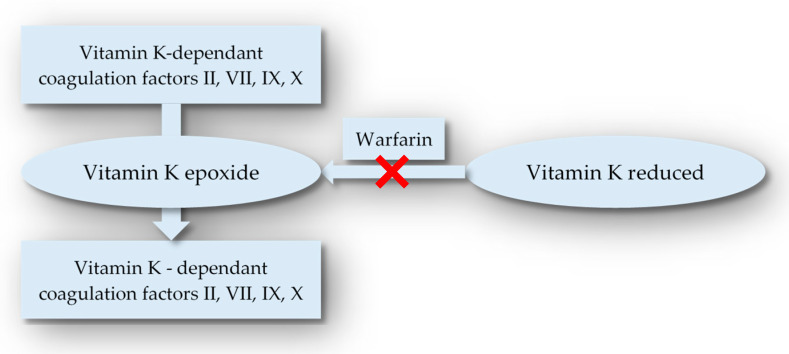
Warfarin mechanism of action.

**Figure 2:**
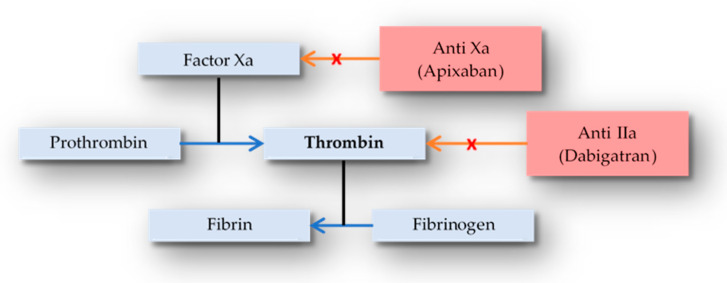
DOAC’s mechanism of action.

The vitamin K-dependent production of coagulation factors II, VII, IX, and X in the liver is inhibited by warfarin (Figure 1 and Figure 2).

Antithrombotic proteins, such as proteins C and S, are also reduced. It has a near-complete oral bioavailability and a maximum plasma concentration of 2 to 6 h. It has a rather small volume of distribution (0.14 L/kg), yet it binds to plasma proteins in substantial amounts (99 percent). Warfarin has a half-life of 20 to 60 h. It is mostly removed by the liver, where it is changed to an inactive metabolite via a cytochrome P450-dependent metabolism and is not eliminated by the kidneys. An INR of 2.0 to 3.0 is considered optimum for the protection of thromboembolic events in the general population, according to AF and VTE recommendations. Unfortunately, there are no particular guidelines for the use of warfarin in individuals with liver disease.

Patients with liver function impairment had a shorter mean duration in the therapeutic window, which has been linked to a two-fold increased risk of major bleeding events [33]. Other variables, such as albumin and glomerular filtration rate, may impact this risk. Warfarin’s safety and effectiveness in preventing thrombotic events have never been studied in a prospective clinical study [10]. In patients with hepatic disease, warfarin may be useful in the treatment of portal vein thrombosis (PVT). Anticoagulant medication with warfarin was found to be effective in a retrospective observational analysis of 28 individuals with PVT. Keeping the INR between 2.0 and 3.0 resulted in higher rates of portal vein revascularization and a lower incidence of recurrent thrombosis.

Even though warfarin has been shown in observational studies of patients with AF and VTE to reduce the risk of thromboembolism in patients with impaired liver function, it has several drawbacks in medical care, including the need for regular INR checking, interactions with nutrition and treatments, phenotypic variation in response, and increasing rates of intracerebral hemorrhage and fatality in comparison with DOACs [10].

Patients with acute liver disease are removed from RCTs, even though DOACs have become the first-choice medication in the treatment and prevention of stroke-systemic thromboembolism and VTE. DOACs have a shorter half-life and are less dependent on hepatic elimination versus warfarin. These pharmacodynamic features make them appealing for use in individuals with liver disease as well.

Apixaban and rivaroxaban are mostly removed by the liver (75 and 65 percent, respectively), with edoxaban (50 percent) and dabigatran (20 percent) following closely behind. These pharmacokinetic qualities can all be influenced to variable degrees by liver disease illness, so they should be used with caution in patients with impaired hepatic function. While liver albumin production is altered, several DOACs have a high plasma protein binding capacity, which can be linked to elevated free drug fraction levels. Apixaban and rivaroxaban are metabolized primarily by cytochrome P450 enzymes, whose activity is lowered by liver disease, whereas dabigatran and edoxaban are processed by cytochrome P450 enzymes to a lesser extent (Table 2). Liver illness reduces the excretion of all DOACs in the bile. Finally, when liver illness is accompanied by hepatorenal syndrome or other kidney disorders coexist, renal clearance of DOACs may be compromised [34].

Anticoagulant therapy should not be used in patients with Child–Pugh Class C, which has a 1-year survival rate of less than 50% without a liver transplant. Patients with portal hypertension, esophageal varices, portal-hypertensive gastropathy, thrombocytopenia, coagulopathy, bleeding risk, decreased drug metabolism, and reduced glomerular filtration rate should be investigated [10].

## 7. The Imbalance between Thrombosis and Bleeding in Liver Disease

Reduced intrinsic anticoagulants and elevated amounts of circulating procoagulants are linked to a higher risk of thrombosis in patients with hepatic disease. In individuals with liver function impairment, a reduction in protein C level and antithrombin synthesis seems to be the main reason for the thrombotic risk. Patients with liver disease are often more likely to experience thrombosis as a result of increased platelet aggregation caused by high von Willebrand (vWF) factor activity and low rates of ADAMTS13 (a disintegrin and metalloprotease with thrombospondin type 1 motif 13), a variable of von Willebrand factor action [35]. They have a greater affinity for both glycoproteins Ib and collagen; therefore, high-molecular-weight multimers are more effective in promoting hemostasis. Other proteases, including plasmin or elastase, can also break down vWF in liver disease. vWF’s multimeric composition is partly influenced by the vWF enzyme that breaks down protease ADAMTS13 [36]. An INR greater than 2.0 was previously thought to protect against VTE; however, more recent observations have disproved this theory. This assertion dates back since studies have shown that even in healthy people, there is a substantial chance risk of developing VTE. It only evaluates the activity of several procoagulant components (FI, FII, FV, FVII, and FX), and not the activity of anticoagulant proteins C and S; therefore, INR does not appear to be a viable tool for monitoring hemostasis in cirrhotic patients.

Specific clotting tests (such as thrombo-elastography) may be able to circumvent and overcome INR’s diagnostic limitations, but they lack proven target values and are thus not commonly employed. Using a PTT test to monitor unfractionated heparin (UFH) treatment presents similar issues to using an INR test to monitor VKAs [22]. All coagulation factors (apart from factor VIII and von Willebrand factor) are generated in the liver; therefore, their plasmatic levels are reduced in liver disease, linked to an increased risk of bleeding. Reduced levels of fibrinogen and factors II, V, VII, and X are reflected in a longer PT, whereas the lower activity of coagulation factors II, V, IX, X, XI, and XII is reflected in a long-activated partial thromboplastin time. Increased fibrinolysis has also been linked to liver disease due to higher levels of tissue plasminogen activator and lower levels of plasmin inhibitor and thrombin-activated fibrinolysis inhibitor [37]. In a compensated cirrhotic patient, the mix of these pro-and anti-coagulant elements creates a delicate equilibrium that can be easily disrupted by any precipitating causes (hepatic decompensation, sepsis, volume status, renal failure, or invasive procedures) and result in thrombosis or bleeding [38,39].

## 8. DOACs—Safety, Efficiency, and Risk of Liver Injury, Gastrointestinal Tolerability

The earliest evidence came through retrospective case studies or small cohort studies that showed DOACs had a lower or equivalent hemorrhagic risk to warfarin in cirrhotic individuals using anticoagulants for various reasons. The confidence interval of these investigations, however, did not always provide a sufficient comparison of thrombotic effects [40]. The American Gastroenterological Association Institute (AGA) suggests using anticoagulation over no anticoagulation in individuals with cirrhosis and atrial fibrillation who have a justification for it. Patients with severe cirrhosis (Child–Pugh class C) and/or low CHA2DS2-VASC scores who place a higher emphasis on avoiding bleeding risk while taking anticoagulants and a lower value on stroke prevention might opt not to take anticoagulation. There was a decrease in mortality in individuals with cirrhosis and atrial fibrillation who were given anticoagulation compared to those who were not (RR, 0.72; 95 percent CI, 0.55–0.94). Patients on DOACs had a decreased risk of nonfatal cerebrovascular accident than those taking warfarin (RR, 0.81; 95 percent CI, 0.73–0.91). Patients who were anticoagulated compared to untreated controls had a greater risk of bleeding (RR, 1.91; 95 percent CI, 1.85–2.26), while the risk was decreased in patients receiving DOACs compared to VKAs (RR, 0.62; 95 percent CI, 0.45–0.85). Similar trends were observed when comparing the incidence of intracranial hemorrhage in patients receiving VKAs to the control group (RR, 3.5; 95 percent confidence interval, 3.30–4.0) with a reduced rate in patients treated with DOACs versus VKAs (RR 0.7; 95 percent confidence interval, 0.58–0.84). In patients with liver cirrhosis and atrial fibrillation with a CHA2DS2-VASC score of 2, the overall advantages of anticoagulation seem to exceed the risk of hemorrhage [30].

In a recent study called “Direct Oral Anticoagulants in Patients with Atrial Fibrillation and Liver Disease”, published by Elsevier in the *Journal of the American College of Cardiology* in 2019, DOACs were compared with warfarin in patients with non-valvular atrial fibrillation and liver disease. The patients were treated with oral anticoagulants (12,778 with warfarin and 24,575 with DOACs), and the outcomes were studied. Ischemic stroke, cerebral and gastrointestinal bleeding, severe bleeding, all-cause fatality, and the composite are all conditions in this situation that can result in death. In patients with AF and significant liver function impairment, DOACs were linked to a decreased incidence of ischemic stroke, intracerebral bleeding, gastrointestinal bleeding, major bleeding, and all-cause mortality when compared to warfarin. In general, DOACs outperformed warfarin in terms of the composite outcome for patients with hepatic diseases, including those with substantial active liver disease. DOAC efficacy and safety were consistently observed in diverse high-risk populations, depending on type and dosing regimen [41].

Concerns regarding DOACs causing liver injury originated from ximelagatran, an oral direct thrombin inhibitor that was widely explored for thromboembolism prophylaxis but was revealed to cause significant liver harm, and it was never approved in the United States. In studies of VTE and stroke protection in AF, there was no difference in the frequency of liver injury comparing warfarin and other DOAC medications. DOAC hepatic safety has been continuously monitored and reported in clinical practice since its approval by the FDA. Elevations of liver enzymes have been linked to all DOACs. During a median follow-up of 14 months, there were seven admissions for liver damage per 1000 person-years in a study of 113,717 patients with AF who were using OACs (50 percent warfarin and 50 percent DOACs). The risk of liver damage was lower in DOAC users than in warfarin users (nine vs. five per thousand person-years).

Dabigatran had the smallest relative risk of liver damage of all the DOACs studied. A meta-analysis of 29 randomized studies comparing DOACs to conventional anticoagulation medication or placebo found that DOACs did not generate liver-related events [10,42].

Many case studies have revealed potentially fatal liver injuries in people taking rivaroxaban or dabigatran. In clinical investigations, the frequency ranged between 0.1 and 1%, and it tended to be lower than enoxaparin or warfarin. The majority of those afflicted were symptomatic, with hepatocellular or mixed liver damage being the most common. This liver injury appears to be associated with all currently available DOACs. Patients should be warned about probable potential symptoms, and these medicines should be stopped if they have significant liver damage [43].

## 9. Management of Bleeding in Patients Who Are Taking DOACs. Therapeutic Methods

The management of bleeding in patients with liver disease who are taking OACs depends on many factors such as the indication for anticoagulation therapy, the underlying thrombotic risk, and the severity of bleeding [10]. There are no differences between the management of bleeding in patients with or without liver disease [44]. Patients with major bleeding events, such as intracranial hemorrhage, gastrointestinal bleeding, muscle bleeds, and extensive hematomas, should be admitted for further evaluation and treatment in an emergency department [45].

According to the 2017 American College of Cardiology expert consensus recommendations, the reversal of anticoagulation may be lifesaving in individuals with life-threatening bleeding despite standard measures. Having said that, life-threatening bleeding in patients with an INR > 2 should be managed with unactivated 4-factor prothrombin complex concentrate (PCC) and intravenous vitamin K [46]. As a result of the similar effectiveness and reduced frequency of side effects, PCC is favored over fresh frozen plasma. Further PCC or vitamin K needs should be determined based on subsequent INR levels and the patient’s clinical state [46].

Specific coagulation tests may aid in determining the amount of anticoagulation in individuals using DOACs. The impact of dabigatran could be measured using thrombin time and ecarin clotting time, whereas the activity of apixaban, rivaroxaban, and edoxaban can be measured using a calibrated anti-factor Xa chromogenic test. These tests, however, are not commonly available and are not always indicated before using reversal drugs [10].

If a patient with liver disease (similar to those without liver disease) experiences major bleeding within 2 h of ingesting an anticoagulant, they should be treated with activated charcoal [10]. Idarucizumab (Praxbind), a completely humanized monoclonal antibody fragment developed to selectively reverse the anticoagulant action of dabigatran, has been approved by the FDA and EMA as a reversal medication for dabigatran, based on the findings of a phase III trial. Antidotes to DOACs, such as andexanet alfa (a factor Xa mimic) and ciraparantag (a neutral small molecule that reverses either factor IIa or Xa inhibitors) are now being investigated as reversal medicines for rivaroxaban and apixaban [2,10]. In clinical studies, PER977 (Perosphere) is being evaluated as a reversal drug for edoxaban, with encouraging preliminary findings [2]. In certain persistently bleeding cases of severe thrombocytopenia, platelet transfusion may be recommended. Proton-pump inhibitors can be used at the same time with a somatostatin analog such as octreotide to reduce portal venous pressure. Additionally, antibiotics should be administered as prophylaxis against spontaneous bacterial peritonitis because it has been shown that administering short-term prophylactic antibiotics in patients with cirrhosis and gastrointestinal hemorrhage increases survival and decreases the rate of bacterial infections [47,48]. The recommended antibiotic is norfloxacin administered orally because it has poor absorption and it is Gram-negative bacteria selective [44]. To enhance platelet function, desmopressin (an endothelial stimulant that raises factor VIII and the von Willebrand factor) can be used especially in patients with liver disease complicated by a hepatorenal syndrome that leads to uremia [10].

Before initiating OACs, all at-risk patients should be checked for esophageal varices and high-risk lesions, according to the American Association for the Study of Liver Diseases’ recommendations [40]. In a study called “Prevention and Management of Gastroesophageal Varices and Variceal Hemorrhage in Cirrhosis”, published by the *American Journal of Gastroenterology* in 2007, there were some recommendations for the management of an acute episode of variceal hemorrhage in patients with cirrhosis. Caution should be taken when resuscitating the blood volume and the goal is maintaining hemodynamic stability and a hemoglobin level of approximately 8 g/dL. Vigorous resuscitation should be avoided with saline solution because this can precipitate the accumulation of fluid at extravascular sites [44]. A meta-analysis of 15 trials comparing emergency sclerotherapy and pharmacological treatment suggested that the first-line treatment of variceal bleeding should be pharmacological because it has a similar efficacy as sclerotherapy [49].

Before commencing OAC therapy, all patients with liver function impairment should be evaluated for alcohol abuse issues and given cessation therapy. Patients should be educated about the potential hazards and advantages of OACs and should be involved in joint decision-making about their usage and selection. Although warfarin has traditionally been the drug of choice for most patients with hepatic conditions needing OACs, DOACs (without dosage modification) might be a safe option for certain individuals with moderate hepatic function impairment (Child–Pugh A). In cirrhotic patients, warfarin is the only OAC advised (Child–Pugh C). When warfarin is not an option, apixaban, dabigatran, or edoxaban may be taken with care in individuals with mild hepatic function impairment (Child–Pugh B) [40].

Although VKAs are frequently thought to be cheaper compared to DOACs, the total cost of VKA treatment must account for expenses linked to therapy management in general. These are examples of routine coagulation monitoring, negative clinical events during medication (such as hemorrhage and thromboembolic events), and non-adherence [50]. The estimated mean number of inpatient days, outpatient visits, and AF-related hospitalizations linked to rivaroxaban are lower in clinical practice than those related to warfarin [51].

## 10. Conclusions and Future Perspectives

According to the available data, NAFLD and NASH are linked to both AF and VTE, showing that these individuals are in a procoagulant condition. Cirrhosis, regardless of the cause, has an unstable hemostatic equilibrium, making anticoagulant therapy difficult [34]. Finally, real-world evidence suggests that DOACs may be administered safely in Child–Pugh A cirrhotic patients and carefully in Child–Pugh B patients, with comparable effectiveness and potentially greater safety than warfarin. Interestingly, rivaroxaban and edoxaban have been used in Child–Pugh B cirrhotic patients without substantial adverse effects in several trials, even though they are contraindicated in these patients by the EMA (European Medicines Agency) and FDA (Food and Drug Administration) [13]. DOACs are a promising alternative for the treatment of AF and VTE in individuals with liver disease, based on growing real-world evidence demonstrating at least equivalent effectiveness and improved protection compared to warfarin. Specific reversal medications for all DOACs are now available for the treatment of bleeding in individuals with liver disease. There is no evidence that a specific DOAC with a superior efficacy/safety profile should be administered more frequently in cirrhotic individuals. RCTs on the use of DOACs in cirrhotic patients are needed [10,31,34]. The CIRROXABAN trial is looking at how rivaroxaban can help patients with liver disease and portal hypertension prevent thrombotic events. There is an unmet need for evidence-based and practical data for guiding anticoagulant therapy [3]. In addition to treatment efficacy and safety, cost-effectiveness is another factor that healthcare professionals evaluate when making treatment decisions.

Although VKAs are frequently thought to have cheaper costs, while the medicine itself is less expensive when compared to DOACs, the total cost of VKA treatment must account for expenses linked to therapy management in general. Routine coagulation monitoring, negative clinical events during medication (such as hemorrhages and thromboembolic events), and non-adherence are all examples. The estimated mean number of inpatient days, outpatient visits, and AF-related hospitalizations linked to rivaroxaban are lower in clinical practice than those related to warfarin [51].

## Figures and Tables

**Table 1:** Oral anticoagulant options related to Child–Pugh classes. Green: no dosage reduction needed, yellow: careful usage, red: contraindication.

	AVKs	DOACs
Child–Pugh A	Warfarin	Dabigatran, Rivaroxaban, Apixaban, Edoxaban, Betrixaban
Child–Pugh B	Warfarin	Dabigatran, Rivaroxaban, Apixaban, Edoxaban
Child–Pugh C	Warfarin	Dabigatran, Rivaroxaban, Apixaban, Edoxaban, Betrixaban

**Table 2:** Oral anticoagulants and their metabolism pathways.

	Hepatic Metabolization (%)	Renal Excretion (%)
Warfarin	100%	0
Apixaban	75%	25%
Rivaroxaban	65%	35%
Edoxaban	50%	50%
Dabigatran	20%	80%

## Data Availability

Not applicable.

## Abbreviations

vitamin K antagonists (VKAs), non-vitamin K antagonists (DOACs), direct oral anticoagulants (DOACs), oral anticoagulants (OACs) nonalcoholic fatty liver disease (NAFLD), nonalcoholic steatohepatitis (NASH), atrial fibrillation (AF), venous thromboembolism (VTE), antiphospholipid syndrome (APS), mitral stenosis (MS), systemic embolism (SE), American College of Cardiology/American Heart Association (ACC/AHA), hazard ratio (HR), gastrointestinal bleeding (GIB), portal vein thrombosis (PVT), international normalized ratio (INR), alanine transaminase (ALT), aspartate transaminase (AST), randomized controlled trials (RCTs), the upper limit of normal (ULN), von Willebrand factor (VWF), unfractionated heparin (UFH), American Gastroenterological Association Institute (AGA), European Medicines Agency (EMA), Food and Drug Administration (FDA).
